# ﻿A new genus, *Sinodromus* gen. nov., with two new species and the first description of the female of *Philodromusguiyang* Long & Yu, 2022 (Arachnida, Araneae, Philodromidae) from China

**DOI:** 10.3897/zookeys.1221.137930

**Published:** 2024-12-20

**Authors:** Zhong-jing Wang, Yan-bin Yao, Zi-ying Tang, Wen-hui Li, Ke-ke Liu, Xiang Xu

**Affiliations:** 1 College of Life Science, Jinggangshan University, Ji’an 343009, Jiangxi, China Jinggangshan University Ji’an China; 2 Jinshan College of Fujian Agriculture and Forestry University, Fuzhou 350007, Fujian, China Jinshan College of Fujian Agriculture and Forestry University Fuzhou China; 3 College of Life Science, Hunan Normal University, Changsha 410081, Hunan, China Hunan Normal University Changsha China

**Keywords:** Distribution, hilly land, running crab spiders, taxonomy

## Abstract

Three species of the spider family Philodromidae are reported from the south of China. A new genus, *Sinodromus***gen. nov.**, is described from Jiangxi, Fujian, and Hunan Provinces. It can be distinguished from other genera of Philodromidae by the tegular apophysis of the palp and the cymbial process, as well as by its uniquely striped abdomen. The type species, *S.fujianensis***sp. nov.**, and a second species, *S.perbrevis***sp. nov.**, are described and illustrated; these species occur in bamboo forests in hilly areas. Additionally, the female of *Philodromusguiyang* Long & Yu, 2022 is described for the first time from Jiangxi and Hunan Provinces. All species are illustrated with SEM micrographs, and their distributions are mapped.

## ﻿Introduction

Philodromidae Thorell, 1870, commonly known as small running crab spiders, is a relatively small but globally distributed spider family consisting of 528 species in 29 genera (WSC 2024). They are free-living, agile spiders commonly found on plants or on the soil surface (Jocqué and Dippenaar-Schoeman 2006). Currently, 59 species in seven genera are recorded or described from China (Li 2020; WSC 2024). The majority of Chinese philodromid spiders have been reported from the Tibetan Plateau in the western part of the country, while other regions of China remain poorly studied (Li and Lin 2016). Thus, there are still many undescribed species of small running crab spiders in the northern and southern parts of China.

While examining philodromid spider material collected in the southern part of China, namely from Hunan, Jiangxi, and Fujian Provinces over the last 10 years, we discovered two undescribed species from one new genus and the first female of *Philodromusguiyang* Long & Yu, 2022. The present paper provides detailed descriptions of these three species.

## ﻿Materials and methods

Specimens were examined using a Jiangnan SZ6100 stereomicroscope with a KUY NICE CCD camera. Male and female copulatory organs in this paper were dissected and examined in 80–85% ethanol. The endogynes were cleaned with pancreatin (Álvarez-Padilla and Hormiga 2007). All specimens were photographed with an Olympus CX43 compound microscope with a KUY NICE CCD camera. For SEM photographs, the specimens were dried under natural conditions, sprayed with gold with a small ion-sputtering apparatus (ETD-2000), or were uncoated, and then photographed with a Zeiss EVO LS15 scanning electron microscope.

All measurements were made using a stereomicroscope (AxioVision SE64 rel. 4.8.3) and are given in millimeters. Leg measurements are given as total length (femur, patella, tibia, metatarsus, tarsus).

Depositories of all specimens examined are abbreviated as:

**ASM-JGSU** Animal Specimen Museum, College of Life Science, Jinggangshan University, Ji’an, China.

**HNU**Hunan Normal University, Changsha, China.

Terminology of the male and female copulatory organs follows Kubcová (2004) and Kastrygina and Kovblyuk (2016). The abbreviations used in the text and figures are:

### ﻿Eyes

**ALE** anterior lateral eye;

**AME** anterior median eye;

**MOA** median ocular area;

**PLE** posterior lateral eye;

**PME** posterior median eye.

### ﻿Male palp

**Con** conductor;

**CP** cymbial process;

**Em** embolus;

**RTA** retrolateral tibial apophysis;

**SD** sperm duct;

**TA** tegular apophysis;

**VTA** ventro-prolateral tibial apophysis.

### ﻿Epigyne

**At** atrium;

## ﻿Taxonomy

### ﻿Family Philodromidae Thorell, 1870

Currently, approximately 43% of philodromid species are known from a single sex and juveniles: 167 of these were described from females, 39 from males, and 21 from juveniles (WSC 2024). There are seven genera reported in China: *Apollophanes* O. Pickard-Cambridge, 1898, *Philodromus* Walckenaer, 1826, *Pulchellodromus* Wunderlich, 2012, *Psellonus* Simon, 1897, *Rhysodromus* Schick, 1965, *Thanatus* C. L. Koch, 1837, and *Tibellus* Simon, 1875 (WSC 2024). Some of these are widely distributed in Asia, America, and Europe, such as *Apollophanespunctipes* (O. Pickard-Cambridge, 1891), *Philodromusemarginatus* (Schrank, 1803), *Pulchellodromusmedius* (O. Pickard-Cambridge, 1872), *Rhysodromusalascensis* (Keyserling, 1884), *Thanatusarcticus* Thorell, 1872, and *Tibellusoblongus* (Walckenaer, 1802) (WSC 2024). Currently, 59 known species in those seven genera above have been reported from China (Li and Lin 2016; WSC 2024). Only three new species have been described from China in the past 10 years (WSC 2024).

#### ﻿Genus *Philodromus* Walckenaer, 1826

##### ﻿ Philodromus guiyang

Taxon classificationAnimaliaAraneaePhilodromidae

Long & Yu, 2022

F578EAB8-2BD4-525C-9314-3F27E2B5D664

**Figure 1.**
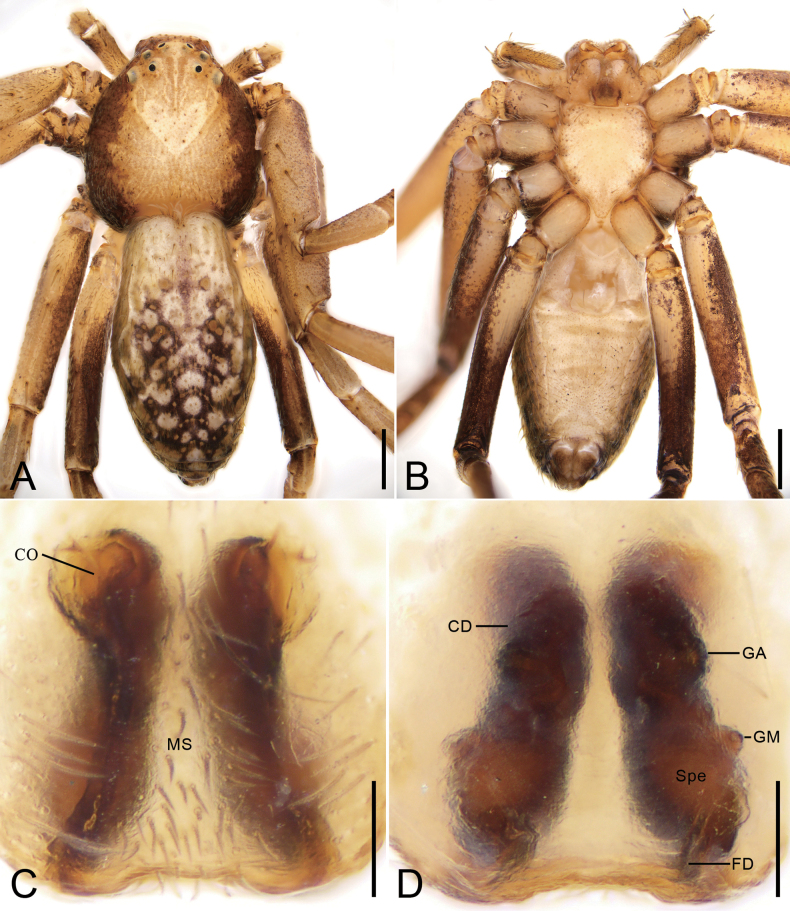
*Philodromusguiyang* Long & Yu, 2022, female **A** habitus, dorsal view **B** same, ventral view **C** epigyne, ventral view **D** vulva, dorsal view. Abbreviations: CD – copulatory duct, CO – copulatory opening, FD – fertilization duct, GA – glandular appendage, GM – glandular mound, MS – median septum, Spe – spermatheca. Scale bars: 0.5 mm (**A, B**); 0.1 mm (**C, D**).

**Figure 2.**
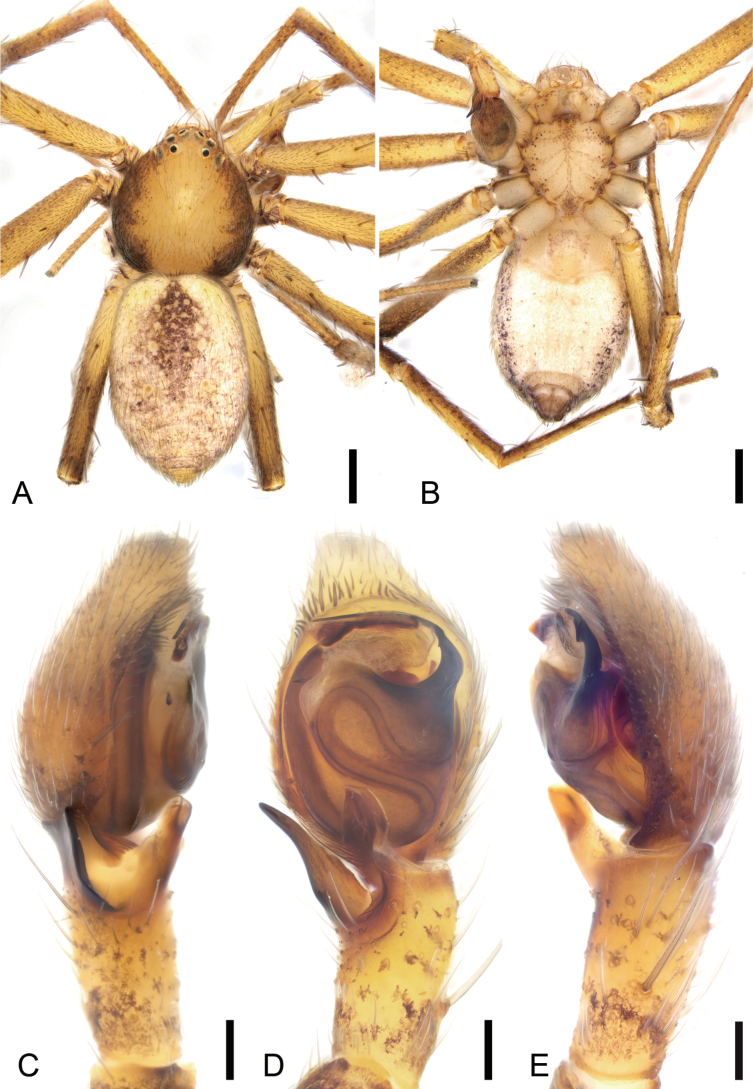
*Philodromusguiyang* Long & Yu, 2022, male **A** habitus, dorsal view **B** same, ventral view **C** right palp, retrolateral view **D** same, ventral view **E** same, prolateral view. Scale bars: 0.5 mm (**A, B**); 0.1 mm (**C–E**).

Figs 1A–D , 2A–E , 8A , 9A, B Common name: 贵阳逍遥蛛

Philodromus guiyang Long & Yu in Long et al. 2022: 118, figs 2A–D, 3A–D (holotype male from Guiyang, Guizhou Province, illustrations examined).

###### Additional material examined.

**China**: Jiangxi Province • 2♂, 5♀, Ji’an City, Jishui County, Dadong Mountain, 27°15'14.71"N, 115°10'50.50"E, 607 m a.s.l., 2 March 2023, K. Liu, Z. Jiang, Z. Deng, X. Chen leg. (20230302, Phi-07, ASM-JGSU) • 1♂, 1♀, Ji’an City, Jinggangshan County Level City, Ciping Town, Jingzhu Mountain, 26°32'45.20"N, 114°06'32.46"E, 1158 m a.s.l., 2 May 2024, Z. Jiang, Z. Wang leg. (20240502, Phi-07, ASM-JGSU) • 1♀, Ciping Town, Huangyangjie Scenic Spot, 26°37'30.33"N, 114°7'8"E, 1384 m a.s.l., 13 August 2024, L. Luo, Y. Yao, Z. Wang leg. (20240813, Phi-07, ASM-JGSU), other data same as previous • 2♀, Shangrao City, Guangfeng District, Tongbo Mountain, Shazi Ridge, 28°09'10.78"N, 118°17'41.31"E, 751 m a.s.l., 11 July 2023, K. Liu, Z. Jiang, C. Li leg. (20230711, Phi-07, ASM-JGSU) • 1♀, Qianshan County, Wangwu Line, Wuyishan Town, near Yu Huizhen Hope Primary School, 27°57'05.51"N, 117°49'12.74"E, 463 m a.s.l., 9 July 2023 (20230709, Phi-07, ASM-JGSU), other data same as previous; Hunan Province • 6♀, Xinning County, Bajiaozhai, Langshan, Bajiaozhai, 26°16.’673N, 110°44.262'E, 839 m a.s.l., 22 July 2015, H. Yin, B. Zhou, J. Gan, Y. Gong, W. Liu, C. Zeng, Z. Chen, B. He, Y. Huang, X. Wu leg. (Phi-07, HNU).

###### Diagnosis.

The female of this species resembles that of *P.subaureolus* Bösenberg & Strand, 1906 (see Yin et al. 2012: 1250, fig. 672b, c) in having widely separated oval spermathecae, but it can be easily separated from it by the broad median septum (vs narrow) and the broad copulatory ducts (vs narrow) (Fig. 1C, D). For the male diagnosis, see Long et al. (2022).

###### Description.

**Female.** Habitus as in Figs 1A, B, 9A, B. Total length 2.95, carapace 1.24 long, 1.3 wide. Eye sizes and interdistances (Fig. 1A): AME 0.06, ALE 0.07, PME 0.07, PLE 0.08, AME–AME 0.17, AME–ALE 0.08, PME–PME 0.31, PME–PLE 0.17, AME–PME 0.18, AME–PLE 0.3, ALE–ALE 0.3, PLE–PLE 0.72, ALE–PLE 0.17. MOA 0.28 long, front width 0.29, back width 0.43. Chelicerae with three promarginal teeth and no retromarginal teeth. Leg measurements: I 5.42 (1.59, 0.7, 1.22, 1.08, 0.83); II 6.26 (1.86, 0.76, 1.56, 1.13, 0.95); III 4.12 (1.45, 0.32, 0.89, 0.88, 0.58); IV 4.47 (1.45, 0.48, 1.04, 0.99, 0.51); spination: I Fe: d6; Ti: d4, p3, r2, v6; Mt: d4, p2, r2, v6; II Fe: d4; Ti: d4, p2, v6; Mt: d4, p4, r3, v6; III Fe: d2, p1; Ti: d4, p2, v4; Mt: d4, p2, r2, v6; IV Fe: d4, p1; Ti: d4, v6; Mt: d4, p2, r3, v6. Abdomen 1.71 long, 1.12 wide.

***Coloration*** (Fig. 1A, B). Carapace white to red-brown, laterally with broad red-brown stripes. Medially with a white V-shaped mark. Chelicerae and endites yellow to brown. Labium brown. Sternum white to brown, laterally with brown spots. Legs white to dark brown, with many dark brown stripes or annulations. Abdomen white to dark brown, with many white spots and yellow muscle sigilla; venter white to yellowish.

***Epigyne*** (Figs 1C, D, 8A). Copulatory openings located at antero-lateral part of epigyne. Median septum broad, sub-posterior part slightly constricted. Copulatory ducts broad, anteriorly curved, posteriorly slightly separated. Glandular appendages slightly protruding, very small, directed laterally. Spermathecae oval, widely separated. Glandular mounds mastoid-like, located on anterolateral part of spermathecae, directed anterolaterally. Fertilization ducts long, more than 2/3 length of spermathecae, directed anterolaterally.

**Male.** See Long et al. (2022) for description; habitus is shown in Fig. 2A, B and the palp is shown in Fig. 2C–E.

###### Remarks.

This species is numerous in subtropical broad-leaved forests. The specimens were collected on shrubs and broad-leaved trees by sieving.

###### Distribution.

Known from Guizhou (Long et al. 2022), Hunan (Fig. 10), and Jiangxi (Fig. 10), China. It may be broadly distributed in southern China.

##### ﻿Genus Sinodromus

Taxon classificationAnimaliaAraneaePhilodromidae

Yao & Liu gen. nov.

352CC0C7-02C6-5D2F-91CD-5D90805EF712

https://zoobank.org/00C43736-D4E3-438C-91E9-5D460295BDF4

###### Type species.

*Sinodromusfujianensis* Yao & Liu, sp. nov.

###### Diagnosis.

**Figure 3.**
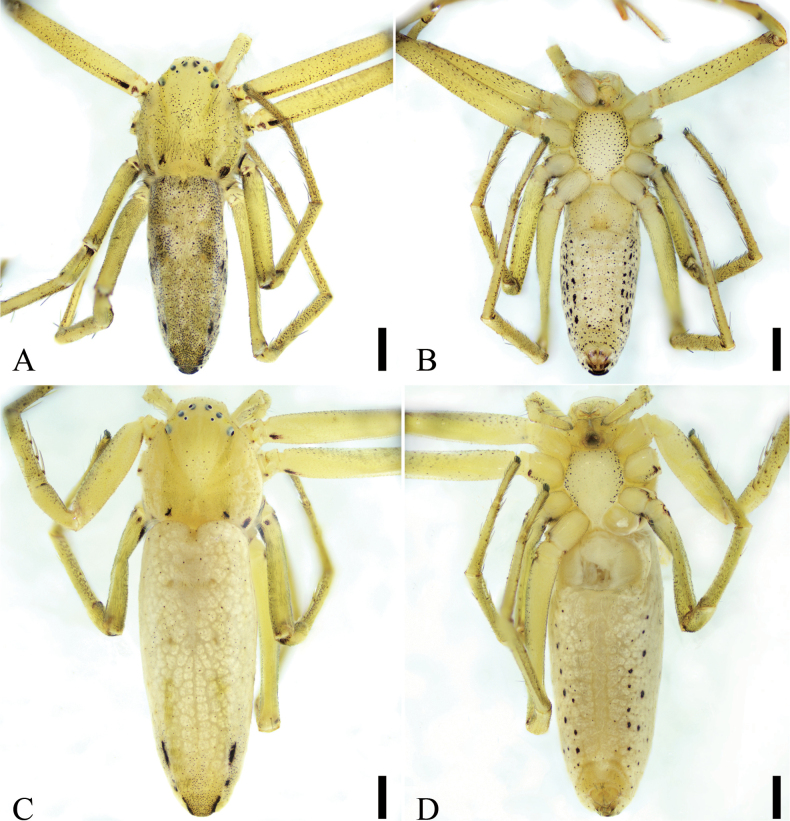
*Sinodromusfujianensis* sp. nov., habitus **A** male holotype, dorsal view **B** same, ventral view **C** female paratype, dorsal view **D** same, ventral view. Scale bars: 0.5 mm.

**Figure 4.**
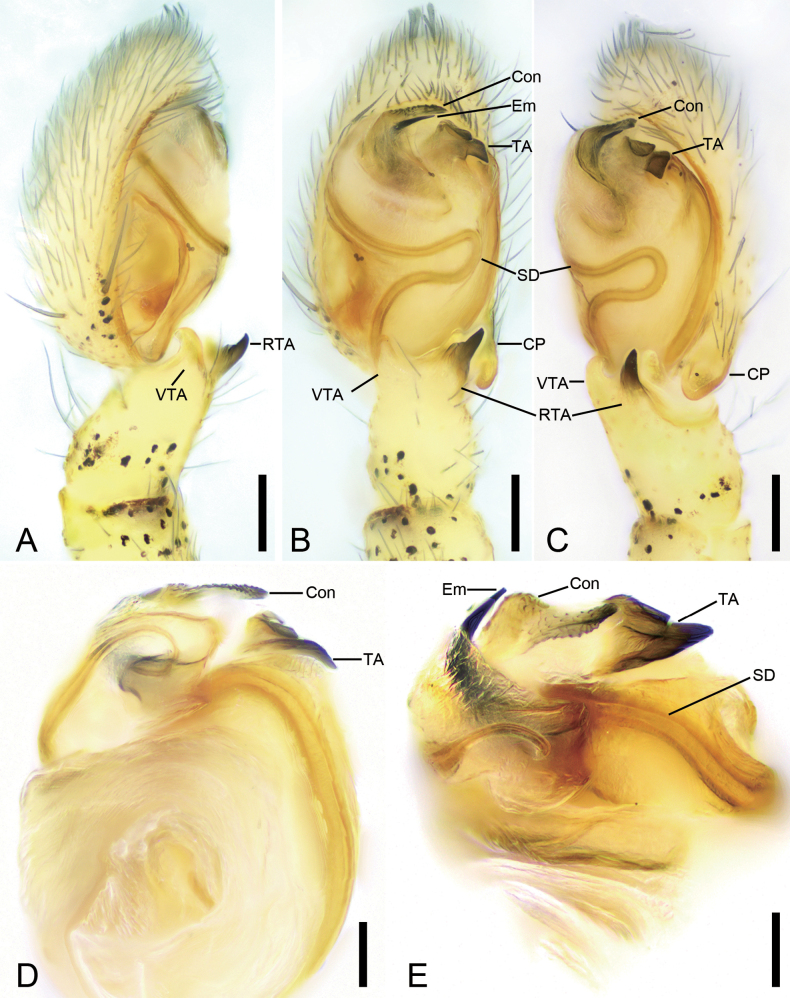
*Sinodromusfujianensis* sp. nov., male palp **A** holotype, prolateral view, slightly ventral **B** same, ventral view **C** same, ventro-retrolateral view **D** paratype, detail of palpal tegulum, posterior view **E** same, ventral view, slightly frontal. Abbreviations: Con – conductor, CP – cymbial process, Em – embolus, RTA – retrolateral tibial apophysis, SD – sperm duct, TA – tegular apophysis, VTA – ventro-prolateral tibial apophysis. Scale bars: 0.1 mm (**A–C**); 0.05 mm (**D, E**).

**Figure 5.**
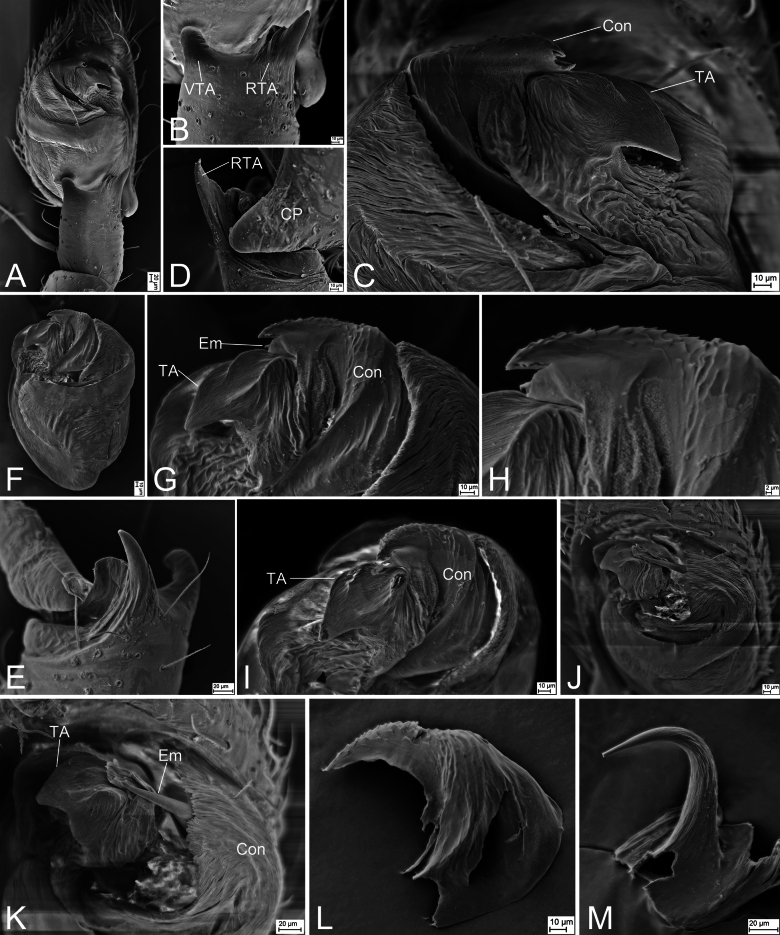
SEMs of *Sinodromusfujianensis* sp. nov., male palp of paratypes **A** left palp, ventral view **B** same, detail of ventro-prolateral and retrolateral tibial apophyses, ventral view **C** same, detail of anterior tegulum, ventral view **D** detail of retrolateral tibial apophysis and cymbial process, retrolateral view **E** right palp, detail of tibial apophyses, prolateral view **F** same, tegulum, ventral view **G** same, detail of anterior tegulum, ventral view **H** same, detail of conductor, ventral view **I** same, detail of anterior tegulum, retrolatero-ventral view **J** same, detail of anterior tegulum, ventral view, slightly frontal **K** same, detail of tegular apophysis and embolic tip after removing part of conductor, ventral view, slightly frontal **L** same, detail of conductor, ventral view **M** same, detail of embolus, ventral view. Abbreviations: Con – conductor, CP – cymbial process Em – embolus, RTA – retrolateral tibial apophysis, TA – tegular apophysis, VTA – ventro-prolateral tibial apophysis.

**Figure 6.**
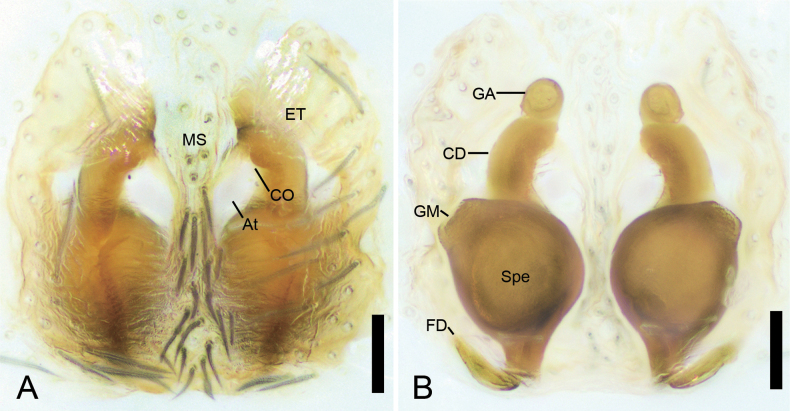
*Sinodromusfujianensis* sp. nov., female epigyne of paratype **A** epigyne, ventral view **B** vulva, dorsal view. Abbreviations: At – atrium, CD – copulatory duct, CO – copulatory opening, ET – epigynal tooth, FD – fertilization duct, GA – glandular appendage, GM – glandular mound, MS – median septum, Spe – spermatheca. Scale bars: 0.05 mm.

**Figure 7.**
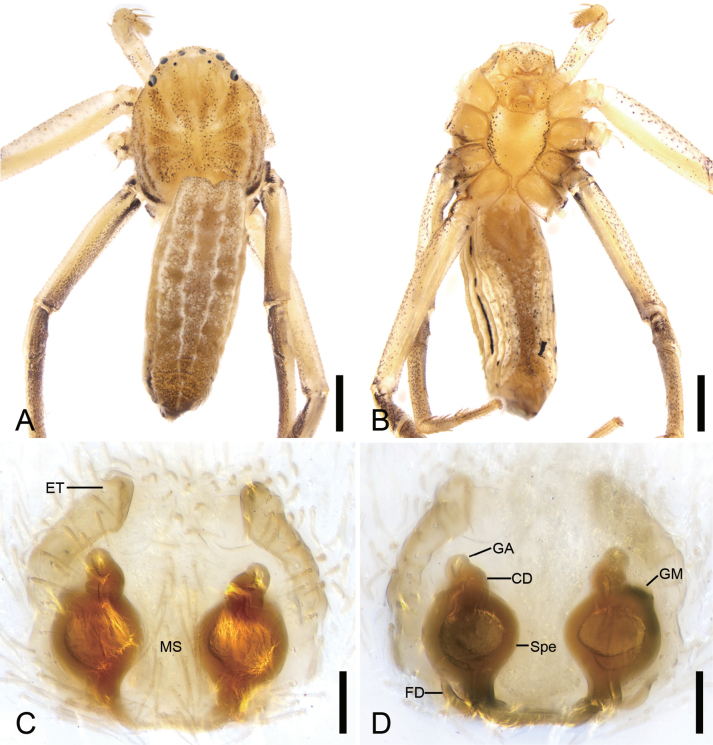
*Sinodromusperbrevis* sp. nov., female holotype **A** habitus, dorsal view **B** same, ventral view **C** epigyne, ventral view **D** vulva, dorsal view. Abbreviations: CD – copulatory duct, ET – epigynal tooth, FD – fertilization duct, GA – glandular appendage, GM – glandular mound, MS – median septum, Spe – spermatheca. Scale bars: 0.5 mm (**A, B**); 0.1 mm (**C, D**).

**Figure 8.**
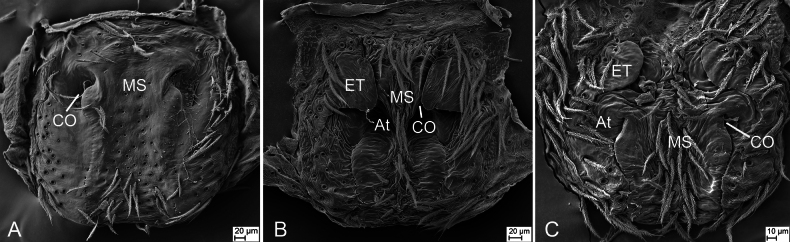
SEM pictures of epigynes, ventral view **A***Philodromusguiyang* Long & Yu, 2022 **B***Sinodromusfujianensis* sp. nov. **C***S.perbrevis* sp. nov.. Abbreviations: At – atrium, CO – copulatory opening, ET – epigynal tooth, MS – median septum.

The new genus is similar to *Tibellus* Simon, 1875 in having a similar habitus (cf. Figs 3–7, 8B, C and Kastrygina and Kovblyuk 2016: figs 1A, 2A, 3A, 4A, 5A, B, 6A, B), but it can be easily distinguished from *Tibellus* (cf. Figs 3–7, 8B, C and Kastrygina and Kovblyuk 2016: figs 1C, G, H, J, K, 2C, G, H, 3F, G, 4C, F, G, 5D, J, K, 6D, F, G) by the very small PME, nearly as long as 1/2 of the AME diameter (vs the large PME as long as AME diameter), the palp with two tibial apophyses (vs one), the presence of a cymbial process (vs absent), the epigyne with a pair of teeth (vs absent), and the relatively thin, tube-shaped copulatory ducts (vs broad). Species of *Sinodromus* gen. nov. also resemble those of *Pulchellodromus* Wunderlich, 2012 in having a blunt cymbium and spine-like RTA (cf. Figs 4A–C, 5A, B, D, E and Lecigne et al. 2019: fig. 3F and Song and Zhu 1997: fig. 134C, D), but the genus can be easily distinguished from *Pulchellodromus* by the slender habitus (vs relatively broad), the male palp with a ventro-prolateral tibial apophysis (vs absent), the well-developed conductor with scaly serrations (vs the undeveloped conductor lacking scaly serrations), and the epigyne with a pair of teeth anterolaterally (vs absent) (cf. Figs 3–7, 8B, C and Lecigne et al. 2019: figs 4F, 5F and Song and Zhu 1997: fig. 134A, B).

###### Description.

Small spiders, body length 2.5–4.5 mm. Male habitus with more black spots than in females. ***Eyes***: AME, ALE, and PLE oval, with relatively large eye cups, PME rounded, smaller than other eyes, with small eye cups, anterior eye row and posterior eye row strongly recurved. Chelicerae with two promarginal teeth and no retromarginal teeth. Broad brown median band present on carapace and abdomen, and white or grey bands present on carapace and abdomen laterally. Abdomen elongate, with a notch anteromedially and pointed at posterior end.

***Male palp***: tibia with two apophyses, ventro-prolateral and retrolateral, both finger-like; cymbium with blunt postero-retrolateral process, directed towards base of retrolateral tibial apophysis; sperm duct slender, curving back on itself, located medially; conductor large, covers embolus, with scaly serrations; tegular apophysis thick and large, slightly sclerotized; embolus spine-like. Epigyne with conspicuous epigynal teeth anterolaterally; median septum triangular; copulatory openings located laterally to median septum; copulatory ducts tube-shaped; spermathecae oval, slightly separated.

###### Species composition.

*S.fujianensis* sp. nov. (type species) and *S.perbrevis* sp. nov.

###### Distribution.

China (Fujian, Hunan, and Jiangxi Provinces; Fig. 10).

###### Etymology.

The genus name is formed from a combination of *sino*- from the Latin “Sinae” referring to China, and -*dromus*, from “Philodromidae”; the gender is masculine.

##### ﻿ Sinodromus fujianensis

Taxon classificationAnimaliaAraneaePhilodromidae

Yao & Liu sp. nov.

41059653-9C0C-5EFD-91D5-09E2C260578B

https://zoobank.org/58446CA8-10D4-4F41-B533-4B3A957649B2

**Figure 9.**
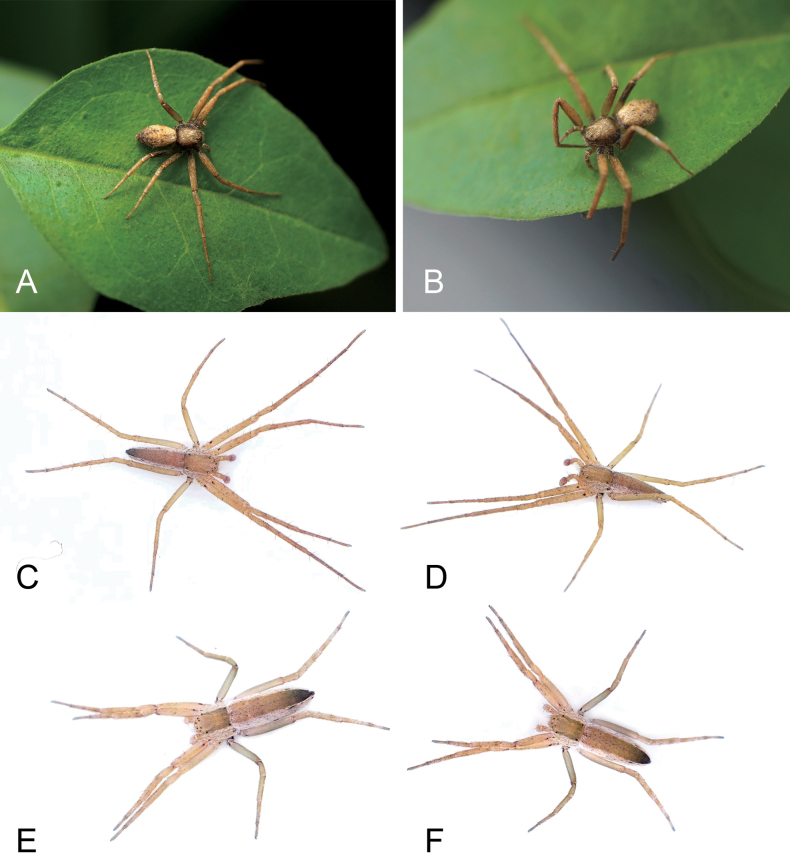
**A, B** living specimen of *Philodromusguiyang* Long & Yu, 2022 **C, D***Sinodromusfujianensis* sp. nov., male **E, F***S.fujianensis*, female.

Figs 3 , 4 , 5 , 6 , 8B , 9C–F Common name: 福建华逍遥蛛

###### Type material.

**China**: Fujian Province: ***Holotype*** • ♂: Fuzhou City, Cangshan District, Jinshan College of Fujian Agriculture and Forestry University, 26°2'21.12"N, 119°19'56.66"E, 24 February 2024, Y. Yao leg. (20240224, Phi-5, ASM-JGSU). ***Paratypes*** • 2♂, 2♀, the same data as the holotype • 2♂, 1♀, Fuzhou City, Yongtai County, Geling Town, Yangxi Village, Tianmen Mountain, 25°49'7.6"N, 119°1'5.07"E, 320 m a.s.l., 23 March 2024, Y. Yao, Q. Wu, and Z. Chen leg. (20240323, Phi-5, ASM-JGSU). Jiangxi Province • 1♀, Ji’an City, Jinggangshan County Level City, Huang’ao Town, Jiebei Group, 26°28'40.8"N, 114°14'16.8"E, 297 m a.s.l., 6 April 2015, Z. Chen, G. Li, K. Liu, Z. Meng, Y. Zhao leg. (20150406, Phi-5, ASM-JGSU).

###### Diagnosis.

Males of the new species are easily distinguished from other philodromid spiders by the following combination of morphological characteristics: (1) the thumb-like retrolateral tibial apophysis with a membranous basal apophysis on the male palpal tibia, (2) the tegular apophysis with several ridges, and (3) the conductor with scale-like serrations (Figs 4A–E, 5A–M). The female resembles that of *Sinodromusperbrevis* sp. nov. in having spermathecae with a short stalk and crescent-shaped fertilization ducts, but it can be separated by the triangular epigynal teeth (vs oval), the copulatory openings located at the mediolateral part of the epigyne (vs anterolateral), and the very short copulatory ducts (vs relatively long) (cf. Figs 6A, B, 8B, 7C, D, 8C).

###### Description.

**Male (holotype)**. Habitus as in Figs 3A, B, 9C, D. Total length 3.83. Carapace medially with dense yellowish-brown setae, laterally with dense white setae, 1.46 long, 1.30 wide. Eye sizes and interdistances (Fig. 3A): AME 0.05, ALE 0.06, PME 0.03, PLE 0.07, AME–AME 0.14, AME–ALE 0.09, PME–PME 0.24, ALE–ALE 0.41, PME–PLE 0.22, PLE–PLE 0.68, ALE–PLE 0.23, AME–PME 0.11, AME–PLE 0.34. MOA 0.15 long, 0.22 front width, 0.30 back width. Chelicerae with two promarginal teeth (proximal larger) and no retromarginal teeth. Leg measurements: I 6.64 (1.91, 0.60, 1.68, 1.53, 0.92); II 8.32 (2.34, 0.82, 2.14, 1.97, 1.05); III 5.18 (1.63, 0.54, 1.22, 1.17, 0.62); IV 6.37 (2.12, 0.65, 1.29, 1.57, 0.74). Leg spination: I Pa: d1, p1, r1; Ti: d2, p2, r2, v5; Mt: d1, p2, r2, v2; II Pa: v1; Ti: d2, p2, r2, v5; Mt: d1, p1, r2, v1; III Pa: v1; Ti: d2, p1, r2, v3; Mt: d2, p2, r3, v3; IV Fe: d2; Pa: d1; Ti: d2, p2, r1; Mt: d2, p2, r3. Abdomen (Fig. 3A, B) medially with dense yellow-brown setae, laterally with dense white setae, 2.47 long, 0.91 wide.

***Coloration*** (Figs 3A, B, 9C, D). Carapace yellow, with many black dots, laterally with broad yellowish stripes, posteriorly with two pairs of black spots. Chelicerae and endites yellow. Labium yellow, with brown spot posteriorly. Sternum shield-like, yellowish, with dense black dots. Legs with many black dots. Abdomen yellowish to yellow, dorsally with dense black dots, medially with broad yellow stripe, laterally with grey stripes; venter with many pairs of black spots bilaterally.

***Palp*** (Figs 4A–E, 5A–M). Tibia with two apophyses, the ventro-prolateral one short, slightly curved dorsally toward posterior part of tegulum, the retrolateral one finger-like, with a membranous basal apophysis. Cymbial process strongly protruding, approaching the base of retrolateral tibial apophysis. Sperm duct thin, curving back on itself, clearly visible from posterior to prolateral part. Tegular apophysis thick, horn-like, with a blunt basal apophysis. Conductor slightly sclerotized, longer than embolus, covers embolus, with many scaly serrations. Embolus short, hook-shaped, tapering to a point.

**Female (paratype).** Habitus as in Figs 3C, D, 9E, F. As in male, except as noted. Total length 5.00. Carapace: 1.50 long, 1.45 wide. Eye sizes and interdistances (Fig. 3C): AME 0.05, ALE 0.05, PME 0.03, PLE 0.06, AME–AME 0.19, AME–ALE 0.11, PME–PME 0.29, ALE–ALE 0.48, PME–PLE 0.24, PLE–PLE 0.80, ALE–PLE 0.22, AME–PME 0.13, AME–PLE 0.36. MOA 0.16 long, 0.28 front width, 0.36 back width. Leg measurements: I 5.22 (1.46, 0.70, 1.31, 1.10, 0.65); II 6.38 (1.76, 0.83, 1.61, 1.39, 0.79); III 4.32 (1.33, 0.50, 1.02, 0.97, 0.50); IV 6.05 (1.97, 0.63, 1.47, 1.33, 0.65). Leg spination: I Fe: d1; Ti: d2, p2, r5, v4; Mt: p3, r3, v3; II Ti: d2, p3, r3, v3; Mt: p3, r3, v2; III Ti: d2, p2, r1, v2; Mt: p3, r1, v3; IV Pa: v1; Ti: d1, p2, r1, v2; Mt: p1, r3, v6. Abdomen 3.50 long, 1.41 wide.

***Coloration*** (Figs 3C, D, 9E, F). Paler than male. Carapace yellowish to yellow, with sparse black dots. Sternum laterally with many black dots. Legs yellowish white, with sparse dark spots. Abdomen with abundant silver spots and sparse black dots on surface.

***Epigyne*** (Figs 6A, B, 8B) slightly longer than wide. Epigynal teeth lamellar, subtriangular, located anterolaterally on epigyne. Atrium small, separated by median septum. Median septum narrow, anteriorly subtriangular. Copulatory openings directed posteriorly, located on the sides of the antero-lateral part of median septum, slightly covered by epigynal teeth. Glandular appendages located at the beginning part of copulatory ducts, directed anteriorly. Copulatory ducts slightly curved, slightly shorter than spermathecae. Spermathecae slightly separated, round, with a short stalk. Glandular mounds slightly protruding, truncate, located on anterolateral part of spermathecae, directed laterally. Fertilization ducts nearly as long as 1/2 of spermathecal width, directed antero-laterally.

###### Biology.

The coloration and habitus are the same as the grassland community from which they are collected and provides them with camouflage.

###### Distribution.

**Figure 10.**
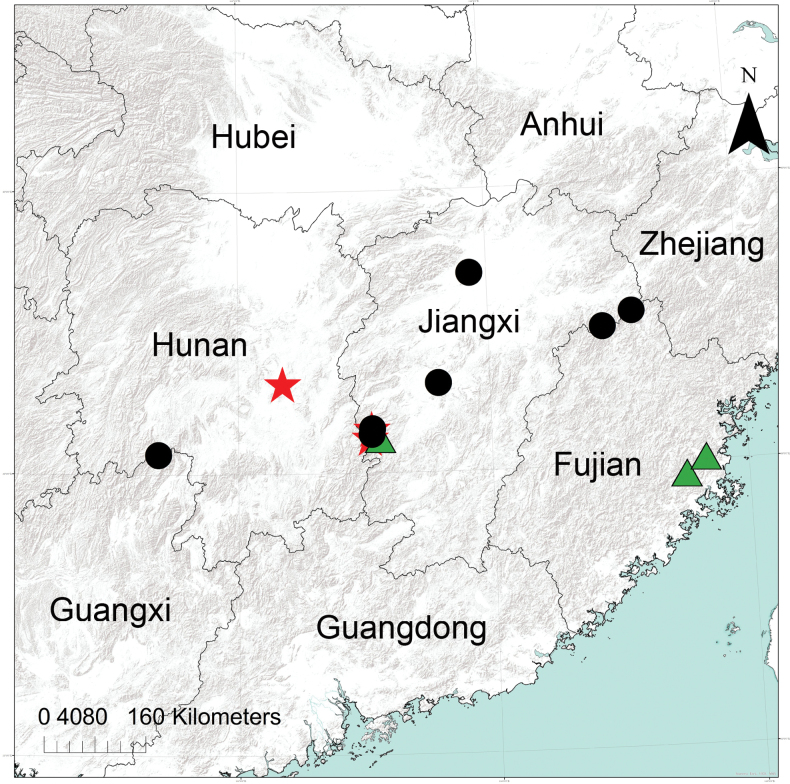
Distributional records of *Philodromusguiyang* Long & Yu, 2022 (black circles), *Sinodromusfujianensis* sp. nov. (green triangles) and *S.perbrevis* sp. nov. (red stars) from China.

Known from the type locality in Fujian Province, as well as Jiangxi Province, China (Fig. 10).

###### Etymology.

The specific name refers to the type locality.

##### ﻿ Sinodromus perbrevis

Taxon classificationAnimaliaAraneaePhilodromidae

Yao & Liu sp. nov.

79A63F9D-5C1F-59B6-9593-D8196B60CB0B

https://zoobank.org/87AD4F3D-EBE1-4D80-A70D-E9540A33B613

Figs 7 , 8C Common name: 短华逍遥蛛

###### Type material.

**China**: Jiangxi Province: ***Holotype*** • ♀, Ji’an City, Jinggangshan County Level City, Ciping Town, Huangyangjie Scenic Spot, 26°37'21.6"N, 114°6'21.6"E, 958 m a.s.l., 5 April 2014, Z. Chen, K. Liu, Z. Meng, Y. Tang, X. Huang leg. (20140405, Phi-06, ASM-JGSU). **Paratypes** • 1♀, Ji’an City, Jinggangshan County Level City, JingZhu Mountain, 26°29'45.6"N, 114°4'44.4"E, 1146 m a.s.l., 20 December 2015, K. Liu, Z. Chen, Z. Meng, Q. Chen, S. Wu, P. Gong leg. (20151220, Phi-06, ASM-JGSU). Hunan Province • 1♀, Hengshan Mountain, 27°16.504'N, 112°42.304'E, 815 m a.s.l., 1–7 May 2007, G. Tang, P. Hu, Q. Wang leg. (Phi-06, HNU) • 1♀, 1 May 2008, Q. Wang leg., other data same as previous (Phi-06, HNU).

###### Diagnosis.

The female of this species can be easily distinguished from *S.fujianensis* sp. nov. by the oval epigynal teeth (vs triangular) and the short copulatory ducts (vs relatively long) (cf. Figs 7C, D, 8C, 6A, B, 8B).

###### Description.

Habitus as in Fig. 7A, B. Total length 3.52. Carapace 1.34 long, 1.21 wide. Eye sizes and interdistances (Fig. 7A): AME 0.04, ALE 0.08, PME 0.03, PLE 0.06, AME–AME 0.18, AME–ALE 0.12, PME–PME 0.28, PME–PLE 0.2, AME–PME 0.12, AME–PLE 0.32, ALE–ALE 0.44, PLE–PLE 0.67, ALE–PLE 0.15. MOA 0.21 long, front width 0.25, back width 0.34. Chelicerae with three promarginal teeth and no retromarginal teeth. Leg (Fig. 7A, B) measurements: I and II missing; III 1.26 (0.42, 0.16, 0.28, 0.25, 0.15); IV 5.3 (1.82, 0.63, 1.18, 1.06, 0.61); spination: III Ti: r2; Mt: d1, r1, v8; IV Ti: v2; Mt: r2, v6. Abdomen (Fig. 7A, B) 2.18 long, 0.79 wide.

***Coloration*** (Fig. 7A, B). Carapace yellow, with three pairs of stripes, each one including many black spots, laterally with dark brown stripes. Chelicerae yellowish, with sparse black spots. Endites and labium yellow, with sparse black spots. Sternum yellowish to yellow, laterally with many black dots. Legs with many small black dots. Abdomen yellowish to brown, dorsally with small dense black dots; venter with a broad brown stripe medially.

***Epigyne*** (Figs 7C, D, 8C) nearly as long as wide. Epigynal teeth lamellar, ear-shaped, located antero-laterally. Atrium moderately large, anteromedially located. Median septum narrow, anteriorly subtriangular. Copulatory openings directed laterally, located on the sides of the antero-lateral part of median septum, not covered by epigynal teeth. Glandular appendages small, mastoid like, located at the origin of the copulatory ducts, directed anterolaterally. Copulatory ducts very short, strongly bent dorsally. Spermathecae widely separated, globular, with a short stalk. Glandular mounds slightly protruding, cap-like, located on antero-lateral part of spermathecae, directed laterally. Fertilization ducts nearly as long as 1/2 of spermathecal width, directed antero-laterally.

**Male.** Unknown.

###### Distribution.

Known from the type locality in Jiangxi, and from Hunan Province, China (Fig. 10).

###### Etymology.

The specific name comes from the Latin word *perbrevis*, referring to the very short copulatory ducts; adjective.

## ﻿Discussion

Currently, with this addition of the two new species, 62 species of philodromids have been classified in eight genera in China. Surprisingly, there are no detailed keys for these genera. The main reasons are: 1) most species of *Thanatus* are known from only a single female (WSC 2024), and it is very difficult to classify the generic characters; 2) the genus *Thanatus* is very large, and the morphological variation within its supposed members is so broad that the assignment of several species to this genus has been questioned (e.g. many species from South China should be re-assigned to the genus *Apollophanes*); 3) the descriptions of *Rhysodromus* and *Tibellus* from China are superficial, and only a few illustrations have been provided (e.g. Song and Zhu 1997; Hu 2001). The new genus has a tegular apophysis (Fig. 5C), which is absent in *Apollophanes* (Dondale and Redner 1975), *Philodromus* (Dondale and Redner 1978), *Pulchellodromus* (Wunderlich 2012), *Psellonus* (Malamel et al. 2019, Lin et al. 2024), *Rhysodromus* (Kastrygina and Kovblyuk 2016), *Thanatus* (Dippenaar-Schoeman et al. 2022), and *Tibellus* (Dippenaar-Schoeman et al. 2022).

Beating as a collecting method has allowed us to simultaneously obtain many specimens from the subtropical forest habitat. The new genus, *Sinodromus* gen. nov., is distributed in the south of China. It is likely that additional species in this genus will be described in the future, extending the distribution.

## Supplementary Material

XML Treatment for Philodromus guiyang

XML Treatment for Sinodromus

XML Treatment for Sinodromus fujianensis

XML Treatment for Sinodromus perbrevis

## ﻿Additional information

### Author ORCIDs

Zhong-jing Wang https://orcid.org/0009-0000-6377-6840

Yan-bin Yao https://orcid.org/0000-0002-2560-9299

Zi-ying Tang https://orcid.org/0009-0006-8513-456X

Wen-hui Li https://orcid.org/0000-0002-8074-9526

Ke-ke Liu https://orcid.org/0000-0001-7822-3667

Xiang Xu https://orcid.org/0000-0001-9485-5373

### Data availability

All of the data that support the findings of this study are available in the main text.
